# Iontophoresis of Biological Macromolecular Drugs

**DOI:** 10.3390/pharmaceutics14030525

**Published:** 2022-02-26

**Authors:** Mahadi Hasan, Anowara Khatun, Kentaro Kogure

**Affiliations:** 1 Division of Animal Disease Model, Research Center for Experimental Modeling of Human Disease, Kanazawa University, Kanazawa 920-8640, Japan; mahadihasan07@gmail.com; 2 Graduate School of Biomedical Sciences, Tokushima University, Shomachi 1, Tokushima 770-8505, Japan; anowarakhatun07@gmail.com

**Keywords:** biological macromolecular drugs, skin barrier, transdermal delivery, iontophoresis, low electricity

## Abstract

Over the last few decades, biological macromolecular drugs (e.g., peptides, proteins, and nucleic acids) have become a significant therapeutic modality for the treatment of various diseases. These drugs are considered superior to small-molecule drugs because of their high specificity and favorable safety profiles. However, such drugs are limited by their low oral bioavailability and short half-lives. Biological macromolecular drugs are typically administrated via invasive methods, e.g., intravenous or subcutaneous injections, which can be painful and induce needle phobia. Noninvasive transdermal delivery is an alternative administration route for the local and systemic delivery of biological macromolecular drugs. However, a challenge with the noninvasive transdermal delivery of biological macromolecular drugs is the outermost layer of the skin, known as the stratum corneum, which is a physical barrier that restricts the entry of extraneous macromolecules. Iontophoresis (IP) relies on the application of a low level of electricity for transdermal drug delivery, in order to facilitate the skin permeation of hydrophilic and charged molecules. The IP of several biological macromolecular drugs has recently been investigated. Herein, we review the IP-mediated noninvasive transdermal delivery of biological macromolecular drugs, their routes of skin permeation, their underlying mechanisms, and their advance applications.

## 1. Introduction

Biological macromolecular drugs (also known as biologics, biomacromolecules, biotechnology drugs) are large and complex molecules composed of sugars, peptides, nucleic acids, or their complex combinations [1,2]. The development of biological macromolecular drugs has garnered significant attention over the last few decades, and such drugs are gradually becoming the leading compounds in the pharmaceutical industry. In 2020, the FDA approved 53 novel therapeutics, including 13 protein drugs and two nucleic acid drugs, across various therapeutic areas [3]. The increasing success of such drugs is likely due to their minimal side effects, high specificity, and endogenous target binding affinity compared to the small-molecule drugs [4].

Despite this recent success, the clinical application of biological macromolecular drugs is associated with a number of challenges [5,6]. Small-molecule drugs are administered orally, which is recognized as the most convenient route to enable patient compliance. On the other hand, biological macromolecules are not suitable for the oral administration, as their large molecular sizes and high degrees of polarity make them impermeable via the intestinal epithelium [7]. Furthermore, these macromolecules are highly susceptible to inactivation in the gastrointestinal tract by several degradation enzymes [8]. The patient-friendly administration route of biological macromolecular drugs has remained primitive for over a decade. To date, these drugs are typically administrated parenterally by intravenous or subcutaneous injection. Side effects are apparent following this invasive route [9], and include pain, the induction of needle phobia, increased risk of infection, and undesirable pharmacokinetics [10,11,12]. Such invasive routes of administration are also time-consuming, and may require a to visit the hospital, which decrease patient compliance. Moreover, the side effects are more prominent when drugs are used to treat chronic medical conditions, which may require long-term repetitive dosing [13]. Considering these limitations, several noninvasive routes have also been investigated, including transdermal, inhalation, buccal and sublingual routes [4,14,15,16]. Inhalation allows drugs to reach the surface of the alveolar epithelium, where then they need to overcome mucociliary clearance, macrophage uptake, and enzymatic degradation in the lungs [17]. On the other hand, the buccal and sublingual routes enable rapid drug uptake through a relatively permeable barrier, but the epithelial surface area is very small, and it is also difficult to maintain a drug delivery system in the mouth [18]. The skin represents an attractive route for the noninvasive delivery of biological macromolecules, owing to its large surface area (1.7 m^2^), which provides a convenient and accessible administration site compared to other routes [19]. Additionally, this route bypasses first-pass hepatic metabolism and avoids drug inactivation by the gastric pH and digestive enzymes in the gastrointestinal tract [20,21]. Furthermore, the transdermal route can allow for the delivery of biological macromolecular drugs at high concentrations, which would be beneficial for the treatment of several skin diseases, including psoriasis and skin cancer. Despite the number of advantages associated with the transdermal administration route, there are some inherent challenges with the noninvasive delivery of biological macromolecular drugs into the skin. In particular, the outermost layer of the skin, namely the stratum corneum (SC), limits the skin permeation of macromolecules [22].

Iontophoresis (IP) refers to the application of a low level of electricity to noninvasive skin permeation technologies [23,24]. IP facilitates the delivery of hydrophilic and charged molecules through the physical layer of the skin by a combined physical and biological mechanism. The IP-mediated noninvasive transdermal delivery of biological macromolecular drugs has recently been investigated. Here, we highlight the recent advances in the use of IP technology for the local delivery of biological macromolecular drugs without the use of drug carriers. The relevant studies of this review were selected from the MEDLINE/PubMed (National Center for Biotechnology Information) and Google Scholar databases using the keywords of this article.

## 2. Challenges of the Noninvasive Transdermal Delivery of Biological Macromolecular Drugs

**Figure 1:**
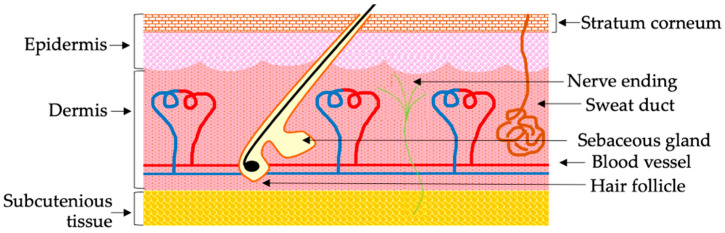
Illustration of mammalian skin. It is composed of epidermis, dermis, and skin appendages (e.g., hair follicles, sweat glands, and sebaceous glands). The outermost layer of the epidermis, known as the stratum corneum, provides the skin barrier function. The other components of the skin include blood vessels and nerves.

The skin is the largest organ in the body, and it provides an ample surface area for drug administration [22]. As such, the noninvasive skin delivery of biological macromolecular drugs represents an alternative to traditional routes of administration. However, the full potential of noninvasive skin delivery has not yet been realized. Mammalian skin exhibits a unique structure, and is composed of two distinct layers known as the epidermis and dermis (Figure 1) [25]. Hair follicles, sweat glands, and sebaceous glands are known as skin appendages, which are derived from invaginated epidermal tissue, and are often rooted into the dermis [25]. The outermost layer of the epidermis, namely the SC, acts as a natural protective barrier to external environments [26]. This layer is typically 10–15 μm thick, and consists of physically non-living keratinocytes, known as corneocytes, that provide the skin’s barrier functions [22,27]. Corneocytes are organized by a protein network (e.g., keratin, filaggrin), and are surrounded by a lipid layer composed of ceramide, cholesterol, and fatty acids [27]. Corneocytes are non-living cells that are continuously replaced in order to maintain the integrity of the SC. This continuous self-renewal of corneocytes mechanically pushes absorbed drugs outside the body [4]. Additionally, the active cellular transport process is non-existent in non-living corneocytes, such that it is not possible to deliver drugs via corneocytes [4]. Besides corneocytes, small lipophilic drugs can diffuse through the lipid layers of the SC. On the other hand, the diffusion of biological macromolecular drugs into the lipid layer is difficult owing to their large molecular sizes and high degrees of polarity [18]. Therefore, under normal conditions, the SC provides a significant barrier (e.g., it is impermeable for hydrophilic molecules with molecular weights >500 Da) for extraneous exposure, making it difficult for macromolecules to penetrate the skin [28]. Below the SC is a viable epidermis layer, which also lacks capillary networks. Even after drugs cross the SC, then they need to reach the dermis layer in order to allow systemic absorption [29]. Taken together, the physicochemical properties of biological macromolecular drugs make it challenging for them to cross the skin barrier.

A number of approaches have been investigated to overcome the skin barrier. These different approaches can be broadly categorized as either chemical or physical enhancement methods [30]. Chemical enhancement is a passive technology that increases the permeability of the SC by altering the lipid structure or by increasing the drug participation on the SC, or a combination of both [31]. Organic solvents (e.g., ethanol), fatty acids (e.g., oleic acid), glycol (e.g., propylene glycol), and surfactants (Tween 80) are commonly used as permeation enhancers [32,33,34]. Recently, the effective ion water-based skin delivery of siRNA has also been reported [35]. Besides these conventional chemical enhancers, encapsulation, or particulate formulation using lipid-based nanoparticles (e.g., liposomes, ethosomes, transferosomes, and niosomes), dendrimers, and polymeric nanoparticles, have also been widely investigated [36,37,38]. These approaches have demonstrated the ability to increase the skin permeation of biological macromolecular drugs up to a certain degree. Some of the common limitations associated with the use of chemical enhancers are skin irritation and the failure to deliver most large macromolecules [39,40]. Moreover, chemical modification may interfere with a drug’s activity, or may result in difficulties in the release of encapsulated drugs [41].

Besides chemical enhancement methods, the application of several physical technologies (e.g., IP, ultrasound, microneedles, electroporation, a pyro-jet injector, and thermal ablation) has garnered significant attention as a means to facilitate the skin permeation of biological macromolecular drugs [42,43,44,45,46,47]. These methods use different types of physical forces that either disrupt or bypass skin barriers. A comparison between each physical method is summarized in Table 1. Recently, the IP-mediated delivery of the TNF-α drug etanercept [48], the ultrasound-mediated delivery of miR-197 [49], and the fractional laser-mediated delivery of small interfering RNA (siRNA) targeting interleukin-6 [50] have all demonstrated promising results against the pathogenesis of psoriasis. Among these mentioned transdermal delivery methods, we emphasized IP technology because of its simple application, which does not require complicated devices [51]. Moreover, IP does not cause cytotoxicity, and can easily be combined with other delivery methods.

## 3. Prospects of IP for the Noninvasive Transdermal Delivery of Biological Macromolecules

IP refers to noninvasive skin permeation technology under the influence of low levels of electricity [52]. The amplitude of the electricity can be varied in each application, but is typically <0.5 mA/cm^2^, which does not induce adverse effects and is recognized as physiologically acceptable [53]. Generally, an IP system contains a positive electrode anode and a negative electrode cathode, a drug reservoir, an electronic controller, and a power source [54]. For IP application, the active electrode containing the drug reservoir is placed on the skin surface, and the circuit is completed by attaching the return electrode containing counter ions adjacent to the active electrode [55]. IP is then achieved by inducing a flow of current from the electrodes to the skin. Here, the flow of electricity provides the driving force for the permeation of drugs across the skin barrier. Charged and hydrophilic molecules of low molecular weight are suitable candidates for IP-mediated delivery [56]. The efficiency and extent of the migration through the skin barrier typically depend on the density and duration of the current’s application and the area of the skin’s surface that remained under the active electrode [57]. The major advantage of IP-mediated drug delivery is its simple application procedure. Furthermore, the drug delivery profile can be customized by modulating the current density and changing the application area [53].

Electrorepulsion and electroosmosis are two physical mechanisms involved in iontophoretic transport [58]. Electrorepulsion is the direct effect of an applied electric field on a charged entity. Examples of electrorepulsion include the migration of positively charged entities into the biological membrane (e.g., skin) under the influence of positively charged electrode anode, or the transfer of negatively charged entities which occurs under the cathode [58]. During electrorepulsion, electron fluxes are transformed into ionic fluxes via the electrode reactions, and ionic transport proceeds through the biological membrane in order to maintain electroneutrality [59]. Electroosmosis, on the other hand, is defined as the convective movement of the solvent by the electric current [60]. The human skin is negatively charged under normal physiological conditions [61]. Therefore, an applied electric field facilitates the migration of positively charged entities across the skin. As a result, electroosmosis is normally directed from the anode to the cathode, and favors the transport of positively charged drugs [60]. Furthermore, anodal electroosmosis also allows for the diffusion of neutral molecules.

In addition to the conventional physical mechanism, Hama et al. investigated the biological effects of IP and the permeation of skin barriers [62]. The authors found that the application of IP on the surface of the skin activates an intracellular signaling pathway that leads to the opening of the intercellular space apparatus, which facilities the migration of liposomes through the skin barrier. In particular, an applied electric field resulted in the cleavage of the gap junctions by decreasing the level of connexin 43 and causing the depolymerization of F-actin associated with tight junctions. These events reduced cell-to-cell interactions and created an intercellular transport shunt, contributing to the migration of substances across the skin barrier. Following this study, Hasan et al. reported that the application of low electric treatment (LET) to cultured cells (similarly to in vivo iontophoresis) induced the rapid and efficient cellular uptake of extraneous macromolecules (e.g., siRNA), unlike electroporation [63]. These authors also studied a wide range of endocytosis inhibitors (e.g., amiloride, filipin, sucrose, and low temperature exposure). When visualized using confocal microscopy, the LET-induced cellular uptake pathway of siRNA was found to be due to endocytosis, whereas endosomes were found to leak macromolecules exhibiting molecular weights < 70,000 Da [64]. Furthermore, these authors identified the specific signaling molecules that contribute to LET-mediated endocytosis; isobaric tags for relative and absolute quantification (iTRAQ) analysis revealed that the application of LET activates numerous signaling molecules (e.g., the up-regulation of the phosphorylation of 139 proteins and the down-regulation of the phosphorylation of 15 proteins) [65]. Among these up-regulated phosphoproteins, it was confirmed that heat shock protein 90, PKC, and the Rho family of small GTPases are major regulators of the LET-mediated cellular uptake pathway.

Based on these findings, Toaro et al. studied the morphology of LET-induced endocytosis [66]. After the LET of PEGylated gold nanoparticles (100 nm, −50 mV), the authors visualized LET-mediated endocytosis by transmission electron microscopy, and found that endosomes containing the gold nanoparticles exhibited tubular, rather than spherical, shapes. This result indicates that the LET-mediated endocytosis is unique and unlike traditional endocytosis. Tubular endocytosis mediated by GTPase regulators associated with focal adhesion kinase-1 (GRAF1) and cdc42 has also been previously reported [67]. Based on the morphological characteristics, LET-mediated endocytosis is suggested to be a kind of GRAF1-and cdc42-dependent endocytosis. Additional details on the biological mechanism of IP are described in our review [53]. Taken together, the applied low electricity of IP provides a driving force and activates an intracellular signaling pathway that cooperatively favors the permeation of skin barriers.

The permeation of skin barriers can be achieved by intracellular, paracellular, and appendageal routes [56]. SC, the horny layer of the skin, consists mainly of non-living corneocytes. Intracellular pathways to permeate the skin barrier require the delivery of substances via corneocytes; however, as non-living cells, corneocytes exhibit no active transport processes. Therefore, the creation of aqueous pores in corneocytes is required in order to initiate intracellular transport. In contrast to the electroporation method, however, it was reported that the application of IP does not create aqueous pores in the SC [68]. Based on this observation, the IP-mediated permeation of the skin barrier likely does not proceed via the intracellular route. The paracellular route refers to the migration of substances through the cells. Generally, small lipophilic molecules (<100 nm) can cross the SC via this route [53]. Recently, it was reported that IP opens intercellular junctions and generates a rapid transport shunt [62]. Therefore, under the influence of IP, biological macromolecular drugs may follow the paracellular route to cross the skin barrier. The appendageal route refers to the delivery of substances via the hair follicles, sweat ducts, and secretory glands [69]. The SC does not exhibit a rigid structure, but instead contains numerous hair follicles, sweat ducts, and secretory glands rooted in the dermis layer [29]. Due to its low water content, the SC exhibits significant electrical resistance. The hair follicles and sweat ducts have much lower electrical resistance compared to the rigid SC [70]. As a result, during IP, electricity preferentially passes through the hair follicles and sweat ducts. Furthermore, the electricity also induces convective solvent flow through these appendageal pathways. Thus, under the influence of an applied electric field, biological macromolecules can migrate through the appendageal pathway to the dermis region, in order to allow for systemic absorption. Taken together, IP mainly induces the transdermal permeation of biological macromolecular drugs via the appendageal pathway, but permeation may also occur, to some extent, via the paracellular route. Besides IP, electroporation is another electricity-assigned drug delivery system. However, electroporation should not be confused with IP. Contrary to IP, electroporation used a high-voltage electric pulse (100–500 Volt) for a micro- to millisecond duration [45]. The high-voltage electric pulse applied to the skin surface creates pores in multilamellar bilayers of SC, and delivers drugs into the skin. Sometimes, membrane damage after electroporation becomes irreversible, which causes apoptosis or necrosis [71]. Although the comparison of the transdermal delivery efficiency of biological macromolecules between IP and electroporation is needed, IP does not cause cytotoxicity, unlike electroporation.

## 4. Recent Advances in the IP-Mediated Transdermal Delivery of Biological Macromolecular Drugs

Molecules that are not suitable for passive diffusion—namely those that are charged, hydrophilic, and exhibit low molecular weights—are ideal for IP-mediated noninvasive delivery [52]. The skin delivery of several the biological macromolecular drugs has recently been achieved using IP without the assistance of a drug carrier. These recent reports are highlighted in this section.

### 4.1. IP-Mediated Intradermal Delivery of siRNA in Skin with Atopic Dermatitis

The noninvasive topical delivery of siRNA is an attractive approach for the treatment of several skin diseases. However, the skin delivery of hydrophilic siRNA by conventional passive diffusion is challenging. Kigasawa K. et al. investigated the IP-mediated skin delivery of unencapsulated siRNA [72]. The authors applied IP to fluorescent-labeled siRNA (e.g., Cy3-labeled siRNA) on ovalbumin-treated atopic dermatitis (AD) model rat skin. Following IP treatment, the fluorescence signal of the siRNA was widely observed in the skin, up to the epidermal and dermal junction. In contrast, in the absence of IP treatment, the fluorescence was only observed on the skin surface. These results suggest that, following application of an electric field, siRNA accumulated mainly in the epidermis, but not the basal layer of the dermis. The overexpression of interleukin-10 (IL-10) is the characteristic feature of AD, which also represents a therapeutic target. The authors also carried out the IP of siRNA against IL-10 on AD skin, and found that siRNA administration via IP significantly suppressed IL-10 mRNA expression by 73%. Taken together, these results suggest that the IP-mediated delivery of siRNA into the skin may be a useful therapeutic strategy for the treatment of AD lesions.

### 4.2. IP-Mediated Transdermal Delivery of Biological Macromolecules for Cancer Immunotherapy

CpG oligodeoxyribonucleotides (CpG-ODN) are single-stranded, short, synthetic DNA molecules containing an unmethylated CpG motif that mimics motifs found in bacterial DNA [73]. Dendritic cells, monocytes, and B-cells take up CpG-ODN via toll-like receptor 9, which results in potent immunostimulatory effects [74]. CpG-ODN monotherapy is advantageous over vaccination because it is not necessary to identify or purify tumor-specific antigens. The skin is the most convenient site for immunization, as numerous antigen-presenting cells—such as epidermal Langerhans cells (LCs) and dermal DCs—reside in the epidermis [75]. Kigasawa et al. investigated the IP-mediated skin delivery of CpG-ODN to induce the activation of an immune response and antitumor activity in B16F1 melanoma-bearing mice [76]. Using fluorescent-labeled CpG-ODN, the authors first confirmed that IP treatment significantly increased CpG-ODN delivery into the epidermis and dermis layers. They also found that the IP-mediated skin delivery of CpG-ODN activated the production of proinflammatory and Th1-type cytokines in the skin, and drained lymph nodes as well. Furthermore, the IP-mediated skin delivery of CpG-ODN significantly suppressed B16F1 tumor growth. Besides CpG-ODN, Toyoda et al. investigated the IP-mediated transdermal delivery of cancer antigen gp100 peptide-loaded nanogels for anticancer vaccination [77]. The authors found that the application of IP delivered the gp100 into the epidermis, and resulted in the activation of Langerhans cells and the suppression of B16F1 tumor growth. Taken together, these results highlight a simple and noninvasive approach for cancer immunotherapy.

### 4.3. Targeting Psoriasis by the IP-Mediated Transdermal Delivery of Biological Macromolecular Drugs

Psoriasis is a chronic immunoinflammatory disease that affects more than 125 million people worldwide, and significantly reduces their quality of life [78]. Although psoriasis initially presents in a benign and noncontagious fashion, its exact pathophysiology remains unknown. Several human and animal studies have revealed that various immune cells (e.g., T-cells, dendritic cells, macrophages, neutrophils, and NK cells) are found in the psoriatic lesions, especially in the dermal and epidermal interface [79,80]. These cells are a significant source of proinflammatory cytokines, especially tumor necrosis factor α (TNF-α), and interleukin-6, which lead to the progression of psoriasis [81]. Although there is no cure for psoriasis, a number of conventional approaches are used to manage psoriasis [82]. Examples of conventional approaches include the topical application of corticosteroids, vitamin D3, combinations of corticosteroids and vitamin D3, and salicylic acid for the treatment of mild psoriasis [83,84], and the systemic application of several nonbiologic immunosuppressive drugs (e.g., methotrexate, cyclosporine) and acitretin (a second-generation retinoid) for moderate or severe psoriasis [85,86]. Recently, the systemic administration of biological macromolecular drugs such as antibodies against TNF-α (e.g., infliximab, adalimumab), interleukin-12 (e.g., ustekinumab), and TNF-α receptor fusion protein (e.g., etanercept) have been used to treat psoriasis when traditional topical and systemic therapies do not achieve sufficient responses [87,88].

In order to overcome the side effects associated with the invasive subcutaneous injection of biological macromolecular drugs, Fukuta et al. investigated the IP-mediated delivery of an antibody and the TNF-α drug etanercept (recombinant human TNF-α receptor: Fc fusion protein) into the skin, and evaluated their therapeutic efficiency against imiquimod (IMQ)-induced psoriasis [48]. The authors applied the IP of FITC-labeled IgG antibodies on the surface of the skin, and observed the fluorescence signal of antibodies that were widely distributed into the epidermis and dermis interface. Repetitive doses of IP and etanercept applied to the surface of IMQ-induced psoriasis skin significantly reduced the expression levels of IL-6 mRNA by 50%. Furthermore, the IP-mediated delivery of etanercept demonstrated the significant suppression of epidermal hyperplasia, which is a characteristic feature of psoriasis. It is interesting to note that the IP-mediated delivery of etanercept provides a greater therapeutic effect compared to the subcutaneous injection of etanercept. This increased therapeutic effect of IP-delivered etanercept compared to subcutaneous injections is likely due to the slow diffusion resulting from IP-mediated delivery.

Based on the results of this study, the authors then performed IP with a tight junction-opening peptide AT1002 analog (Arg-Arg-Arg-Gly-Gly-Phe-Cys-Ile-Gly-Arg-Leu) [89]. It has been reported that AT1002 assists in the skin permeation of topically applied drugs [90]. Therefore, the combination of IP with AT1002 was anticipated to induce the more efficient transdermal permeation of biological macromolecular drugs across hyperproliferative psoriatic skin. In addition to etanercept, the authors also evaluated NF-κB decoy ODN as a biological macromolecular drug, and applied the combination system of IP and the AT1002 analog onto psoriasis skin. The activation of NF-κB pathways is the hallmark of psoriasis, and results in the production of excessive inflammatory cytokines (e.g., TNF-α, IL-6, IL-17), which leads to the progression of psoriasis [91]. Therefore, the NF-κB decoy ODN-mediated selective inhibition of NF-κB signaling represents a promising therapeutic strategy for the treatment of psoriasis. Interestingly, the authors found that a single dose of NF-κB decoy ODN delivered via the combination of IP and the AT1002 analog peptide showed improved therapeutic effects against psoriasis, which significantly suppressed epidermal hyperplasia as well as the production of TNF-α and IL-6 mRNA. Taken together, the combined system results in a cooperative effect that efficiently overcomes the thickened psoriatic skin barrier and enables the transdermal delivery of biological macromolecular drugs for the treatment of psoriasis.

### 4.4. IP-Mediated Transdermal Delivery of Cetuximab

Squamous cell carcinoma (SCC) is a non-melanoma skin cancer originating from keratinocytes in the viable epidermis [92]. Epidermal growth factor (EGF) receptor overexpression is a hallmark of SSC [93]. Cetuximab is a recombinant human/mouse chimeric monoclonal antibody that binds specifically to the extracellular domain of the human epidermal growth factor receptor (EGFR) [94]. The FDA approved cetuximab to treat SCC and colorectal cancer. Lapteva et al. evaluated the IP-mediated skin delivery of cetuximab, and found that IP treatment induced the skin permeation of cetuximab [95]. Therapeutic concentrations of cetuximab are delivered into the viable epidermis after the application of IP (0.5 mA/cm^2^) for 1 h, and after 4 h for the upper dermis, and after 8 h for the lower dermis. Moreover, the authors also found that IP application enables cetuximab delivery via both the intercellular and follicular routes. Taken together, this study demonstrates the feasibility of IP-mediated efficient antibody delivery into the skin.

### 4.5. IP-Mediated Transdermal Delivery of Biologically Active Human Basic Fibroblast Growth Factor (hbFGF)

hbFGF belongs to a large family of fibroblast growth factors involved in the proliferation, differentiation, migration, and survival of different types of cells [96]. hbFGF has shown promising results for the treatment of various dermatological conditions (e.g., skin ulcers and burns in both adult and pediatric patients) [97,98]. Dubey et al. investigated the IP-mediated transdermal delivery of hbFGF across the skin barrier [99]. Following IP of fluorescent-labeled hbFGF, the authors confirmed the subsequent delivery and distribution of hbFGF into the epidermis and dermis layers by confocal laser scanning microscopy. In addition, the skin permeation and deposition of hbFGF were evaluated following an enzyme-linked immunosorbent assay. Among three applied electrical densities (e.g., 0.15 mA/cm^2^, 0.3 mA/cm^2^, and 0.5 mA/cm^2^), it was found that the IP-mediated permeation of hbFGF was superior at 0.3 mA/cm^2^. Using HFF and NIH3T3 cell proliferation assays, the authors further confirmed that the hbFGF retained its biological activity following IP treatment.

### 4.6. Application of IP onto Internal Organs

Although IP is a noninvasive transdermal drug delivery technology, a recent study reported the application of IP to the liver [100]. Liver fibrosis and steatosis gradually develop into liver cirrhosis, a leading cause of mortality and morbidity worldwide. Currently, liver transplantation is the only effective therapy for liver cirrhosis; however, transplantation is often limited because of the lack of availability of liver grafts. In this study, the authors succeeded in the local delivery of siRNA into the liver following the application of IP. The heat shock protein 47 (HSP47) gene is known to be up-regulated in fibrosis, and it assists with collagen deposition in fibrotic liver, while resistin is associated with lipid accumulation, and is known to be abundantly present in liver cells [101,102,103]. The authors found that the IP-mediated delivery of siRNA against HSP47 in CCl_4_-induced fibrosis mouse liver, and of siRNA against resistin in the liver of KK-A^y^ obesity model mice significantly suppressed the gene expression and ameliorated the pathological phenotypes of liver fibrosis and fatty liver disease, respectively. Furthermore, the authors also demonstrated the significant knockdown of the Pdx-1 gene following the application of IP with anti-Pdx-1 siRNA onto the pancreas. Although IP application to internal organs requires a surgical incision, it can induce the specific local delivery of siRNA into the disease-affected tissues, thus eliminating systemic toxicity and non-specific distribution to other organs. Laparoscopic liver resection (LLR) is now gaining popularity [104]. This procedure offers a minimally invasive, safe and effective surgical approach for the liver [104]. In addition, laparoscopic surgery has also shown promising results in the pancreas [105]. In the future, IP application technologies in combination with laparoscopic surgical devices (e.g., a robotic surgical system) may allow for the minimally invasive delivery of biological macromolecular drugs into internal organs for the treatment of fatal diseases. Table 2 summarizes the use of IP for delivering biological macromolecular drugs reviewed in Section 4.

## 5. Delivery of Biological Macromolecules by the Combined Application of IP and Other Permeation Techniques

Besides IP, other permeation techniques such as liposomes, polymeric nanocarriers, ionic liquids, skin-penetrating peptides, microneedles, and ultrasound are reported to induce the topical delivery of biological macromolecules [106,107]. Therefore, the cooperative effect of the application of IP in combination with such enhancement techniques should improve the delivery efficacy of biological macromolecules. Yang et al. developed a smartphone-powered iontophoresis-microneedle array patch (IMAP) which combines IP and nanovesicles [108]. Microneedles of IMAP create microchannels in the skin while the application of IP delivers nanovesicles through the microchannel. The combined effect of microneedles and the IP of IMAP significantly improved insulin-loaded nanovesicle delivery, and showed an excellent hypoglycemic effect in a type 1 diabetic rat model. Noh et al. reported the application of IP with a new type of microneedle called Tappy Tok Tok^®^ that has a diameter similar to the thickness of hair follicles [109]. After 1 min of pretreatment with the microneedles, the authors applied the IP of recombinant human growth hormone (hGH). They found that the combined application increased the transdermal delivery of hGH nearly sixfold compared to single applications of microneedles or IP.

Kajimoto et al. investigated the combined application of IP with liposomes for the transfollicular delivery of insulin [29]. This study found that the IP of insulin-encapsulated cationic liposomes composed of DOTAP/egg phosphatidylcholine (EPC)/cholesterol (Chol) at a molar ratio of 2:2:1 achieved a greater delivery depth via the hair follicles. Consequently, it showed excellent glucose regulation in which low blood glucose levels were maintained for up to 24 h. Moreover, IP-mediated transdermal delivery of liposome-encapsulated antioxidative enzyme superoxide dismutase against UV-induced skin damage, and STAT3 siRNA against melanoma have been investigated [110,111]. In both cases, the combined application of IP with liposomes showed an improved therapeutic effect compared to their corresponding single applications. Here, the mentioned studies used cationic liposomes. Therefore, such an improved therapeutic effect was observed due to the synergistic effect of liposomes and IP, where liposomes may increase the drug participation on the skin and assist the IP-mediated permeation of SC. In addition to microneedles and liposomes, it has also been reported that the application of IP with a chemical penetration enhancer (e.g., limonene/ethanol), and dendrimer enables the transdermal delivery of the biological macromolecules summarized in Table 3 [112,113].

## 6. Limitations of the IP-mediated Delivery of Biological Macromolecular Drugs

IP uses a weak electrical current density (<0.5 mA/cm^2^) which pushes drug molecules into the appendageal or intercellular routes, rather than the direct breakdown of SC. Biological macromolecules, on the other hand, exhibit large molecular sizes. Therefore, effective IP-mediated transdermal permeation and the achievement of the therapeutic concentration of such macromolecules are challenging. The successful delivery of macromolecules with a molecular weight >15 kDa is limited by IP [23]. Additionally, the delivery efficiency may vary based on each macromolecule’s physicochemical properties (e.g., solubility, stability), which needs to be investigated. The application of IP also has several safety issues. Although the amplitude of electricity of IP is physiologically acceptable, it can elicit several effects on the skin. Sometimes, patients may experience skin irritation, a numb feeling, itching, and erythema [23]. Moreover, the selection of inappropriate electrodes or placing them in defective skin, a longer duration of application, and a strong current density may increase the risk of burns [114].

## 7. Clinical Status and Commercialization of the IP of Biological Macromolecular Drugs

Biological macromolecular drug development is a rapidly growing field that has increased the number of drugs in recent years. However, the clinical application of the IP of such drugs remains at the laboratory level. To date, the transdermal IP of fentanyl, lidocaine, and sumatriptan has been approved by The Food and Drug Administration (FDA) in order to manage post-operative pain, the induction of local anesthesia, and the treatment of migraines, respectively. Additionally, clinical trials of several studies related to iontophoresis have been reported [115], e.g., the IP-mediated delivery of methotrexate for the treatment of palmar psoriasis [116], the IP of treprostinil on a finger pad to improve the blood flow in patients with systemic sclerosis [117], and the transdermal IP of neostigmine/glycopyrrolate to initiate bowel evacuation in patients with spinal cord injury [118]. However, these studies reported on small-molecule drugs. Some challenges need to be overcome for the clinical translation and commercialization of the IP of biological macromolecules. For example, the delivery efficacy of IP of macromolecules needs to be improved. The development of a macromolecule-embedded IP patch that enables the combined application of IP with other enhancement techniques may be an excellent choice to improve it. Most of the commercial IP devices in the market are expensive and bulky, and require external power supplies [119]. Therefore, cost-effective, smart IP devices are needed. Moreover, in order to ensure the clinical quality of IP products (e.g., devices, electrodes, and IP patches), the guidelines of several regulatory authorities—likely the FDA, European Medical Agency (EMA), and the Ministry of Health, Labor and Welfare (MHLW) of Japan—should be followed for the corresponding region.

## 8. Conclusions

The development of biological macromolecular drugs has been continuously expanding in recent years, as noninvasive routes of administration are preferable for these types of drugs. IP has garnered significant interest in the noninvasive skin delivery of biological macromolecular drugs owing to its simple application procedures. In this review, we discussed the potential applications of IP, and its underlying mechanism, to overcome the challenges associated with the noninvasive transdermal delivery of biological macromolecules. Various studies have demonstrated the successful and effective IP-mediated transdermal delivery of biological macromolecular drugs. However, their therapeutic effects have been mainly limited to skin diseases, although a recent study applied IP to the internal organs (e.g., liver, pancreas). Further investigations and efforts are needed in order to develop a versatile IP application system for the noninvasive delivery of biological macromolecular drugs.

## Figures and Tables

**Table 1:** Comparison between different physical methods for the transdermal delivery of biological macromolecular drugs.

Methods & References	Driving Forces	Advantages	Disadvantages
Iontophoresis [42]	Weak electric current (<0.5 mA/cm^2^).	Effective for delivery of small molecules and large macromolecules. Easy application procedure and self-administration is possible. Does not cause cell damage.	Skin irritation may occur. Incorrect choice of electrodes may have the risk of burn.
Electroporation [43]	High-voltage electric pulses (30–500 Volt) for micro to milli second.	Induces rapid drug delivery. Effective and reproducible.	Causes cell damage Application is limited to a small area. High electric voltage may affect drug molecules.
Ultrasound [44]	High frequency ultrasound (0.7–3 MHz), low frequency ultrasound (20–100 kHz).	Enhances skin permeability. Therapeutic concentration of small and large macromolecules can be delivered.	Time consuming. Causes skin irritation and risk of burns. SC is broken for effective delivery.
Microneedles [45]	Mechanically 100–1000 μm needles penetrate through the SC.	Induces direct delivery through SC. Skin area can be customized for drug delivery.	Minimally invasive. Allergy or inflammation may be caused at the administration site. Limited dosing is possible due to the small size of microneedles. Sometimes needles can be broken or remained in the skin.
Pyro jet injector [46]	High velocity liquid jet (100–200 m/s).	Effective for vaccination. Reduces needle phobia.	Induces pain. Sometimes adverse reaction may occur at the injection site.
Thermal ablation [47]	Microsecond heat pulse selectively removes SC.	Increases permeability of SC. Enables transdermal delivery of small molecules and macromolecules.	Skin structure is changed, or SC is broken. Use of high heat pulse is a subject of concern and inappropriate instrumentation my cause burns.

**Table 2:** A brief explanation of the IP-mediated delivery of biological macromolecular drugs, and the important outcomes.

Biological Macromolecular Drugs	Dose of IP	Model	Important Outcome of the Study	Reference
Anti-IL-10 siRNA	0.3 mA/cm^2^, for 1 h	Ovalbumin-induced atopic dermatitis rat	IP-mediated delivery of siRNA into the epidermis significantly reduced IL-10 mRNA expression.	[72]
CpG-ODN	0.3 mA/cm^2^, for 1 h	B16F1 melanoma bearing mouse	Transdermal delivery of CpG-ODN by IP induced pro-inflammatory cytokine production and inhibited the tumor growth.	[76]
GP 100	0.4 mA/cm^2^, 1 h	B16F1 melanoma bearing mouse	IP-mediated transdermal delivery of GP 100 activated immune responses and inhibited the tumor growth.	[77]
NF-κB decoy ODN	0.34 mA/cm^2^, 1 h	IMQ-induced psoriasis rat	IP-mediated transdermal delivery of NF-κB decoy ODN significantly reduced proinflammatory cytokine production and reduced epidermal hyperplasia.	[89]
TNF-α drug etanercept	0.34 mA/cm^2^, 1 h	IMQ-induced psoriasis rat	IP-mediated delivery of TNF-α drug etanercept into the epidermis significantly reduced epidermal hyperplasia.	[48]
Cetuximab	0.5 mA/cm^2^, 2, 4, 8 h	Porcine skin	IP induced transdermal permeation of cetuximab.	[95]
hbFGF	0.15, 0.3, 0.5 mA/cm^2^, 8 h	Porcine skin, Human skin	IP induced transdermal delivery of hbRGF.	[99]
Anti-HSP47 siRNA	0.34 mA/cm^2^, 30 min	CCl_4_-induced fibrosis mice	IP employed hepatic delivery of siRNA and significantly suppressed HSP47 expression leading to the reduction of collagen deposition in fibrotic liver.	[100]
Anti-resistin siRNA	0.34 mA/cm^2^, 30 min	KKA^y^ obesity model mice	IP-mediated hepatic delivery of anti-resistin siRNA significantly reduced lipid accumulation in liver.	[100]
Anti-Pdx-1 siRNA	0.34 mA/cm^2^, 30 min	BALB/c Mice	IP employed pancreatic delivery of siRNA and induced significant RNA interference effect.	[100]

**Table 3:** Combined application of IP with other permeation techniques.

Biological Macromolecules	Method Combined with IP	Model/IP Dose	Outcome	References
Insulin	Microneedles	Type 1 diabetic rat (In vivo)/Microneedle array/1 mA, 1 h	Induced controlled insulin delivery and significant hypoglycemic effect.	[108]
hGH	Microneedles	Rat Skin (in vitro)/ 0.5 mA/cm^2^, 4 h	Increased transdermal delivery of hGH as of 6-fold compared to single applications.	[109]
Insulin	Liposomes DOTAP/EPC/Chol = 2:2:1 (molar ratio)	Diabetic Rats (In vivo)/ 0.45 mA/cm^2^, 1 h	Gradually reduced blood glucose level up to 24 h.	[29]
superoxide dismutase	Liposomes DOTAP/EPC/Chol = 2:2:1 (molar ratio)	UV irradiated Rats (In vivo)/0.45 mA/cm^2^, 1 h	Suppressed skin damage-associated marker.	[110]
STAT3 siRNA with curcumin	Liposomes DOTAP/DOPE/C6 Ceramide/Sodium Cholate = 50:30:10:10 (*w*/*w*)	Melanoma bearing mice (In vivo)/0.47 mA/cm^2^, 2 h	Exhibited greater tumor suppression compared to single applications.	[111]
Antisense oligonucleotide	Chemical enhancer (limonene/ethanol (1:1))	Pig ear skin (In vitro)/ 1.25 mA/cm^2^, 4 h	Synergistic effect increased transdermal delivery of antisense oligonucleotide.	[112]
Antisense oligonucleotide	PAMAM dendrimer	Skin cancer mice (In vivo)/0.5 mA/cm^2^, 2 h	Combined application suppressed 45% of tumor volume.	[113]

## Data Availability

Not applicable.
